# A hybrid bipy–NHC ligand for the construction of group 11 mixed-metal bimetallic complexes†

**DOI:** 10.1039/d1ra06581e

**Published:** 2021-10-21

**Authors:** Benson M. Kariuki, James A. Platts, Paul D. Newman

**Affiliations:** School of Chemistry, Cardiff University Park Place Cardiff CF10 3AT Wales UK; Cardiff Catalysis Institute, School of Chemistry, Cardiff University Park Place Cardiff CF10 3AT Wales UK newmanp1@cardiff.ac.uk

## Abstract

An asymmetric bipy/NHC ligand L has been used to construct Au/Au, Au/Ag and Au/Cu bimetallic complexes through prior coordination of the NHC to Au(i) and subsequent introduction of the second group 11 metal ion at the bipy donor of the hybrid ligand. The complex [Au(κ^C^-L)_2_]BF_4,_1, has been used as the precursor for the formation of [AuAg(κ-*C*^Au^,κ^2^-*N*,*N*′^Ag^-1)_2_](BF_4_)_2_, 2a, [AuCu(κ-*C*^Au^,κ^2^-*N*,*N*′^Cu^-1)_2_](BF_4_)_2_, 2b and [AuAu′(κ-C^Au/Au′^,κ^1^-N^Au/Au′^-1)_2_](BF_4_)_2_, 3.

## Abstract 2

A ditopic bipy–NHC ligand has been used to construct hetero-bimetallic complexes of the monovalent group 11 metals.

## Graphical abstract

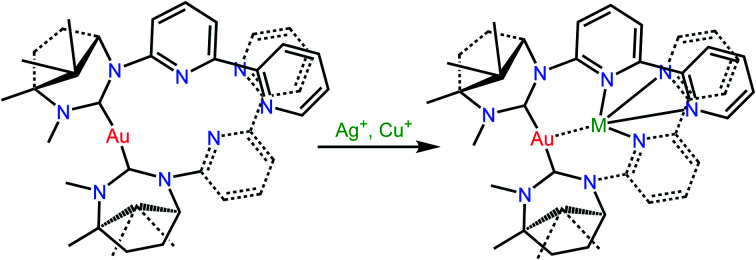

## Introduction

Bimetallic complexes have many interesting applications in fields such as medicinal chemistry, materials science and catalysis.^1^ Whilst homo-metallic systems are common, the inclusion of two disparate metal ions can give complexes with wider-reaching capabilities. Controlled formation of such systems is often challenging as the simultaneous inclusion of both metals is not usually possible and each unique ion has to be introduced sequentially. One method to enable this is to utilise ligands with two (or more) distinct binding sites to selectively accommodate the two metal ions.^2^ This can be achieved by exploiting the preference of certain metal ions for certain donors or by having a masked function that enables discrete inclusion of a first metal before release of the masked donor to sequester the second metal ion. Imidazolium or amidinium salts are often employed as protected forms of N-heterocyclic carbenes (NHCs) and hence these, in combination with other donors, are a popular choice for the construction of both homo- and hetero-bimetallic compounds.^3–10^

Gold NHC complexes are well established systems for various applications including their use as metallodrugs^11^ or in catalysis.^12^ The nature of the NHC ranges from traditional 5-membered systems^11,12^ through so-called expanded-ring NHCs^12*b*,13^ to more sophisticated frameworks.^14^ Furthermore, NHC-based ligands have been utilised for the controlled formation of bi- or multimetallic complexes of the group 11 metal ions where aurophilic interactions are present.^3–10,15^ NHC donors derived from the bicyclic camphor framework have been prepared by ourselves^16^ and the group of Wilhelm^17^ for, in particular, the development of heterodonor ligands. We have extended this chemistry to the synthesis of an amidinium-functionalised bipy derivative with the express intention of forming mixed metal bimetallics and we detail our initial studies here.

## Results and discussion

**Scheme 1:**
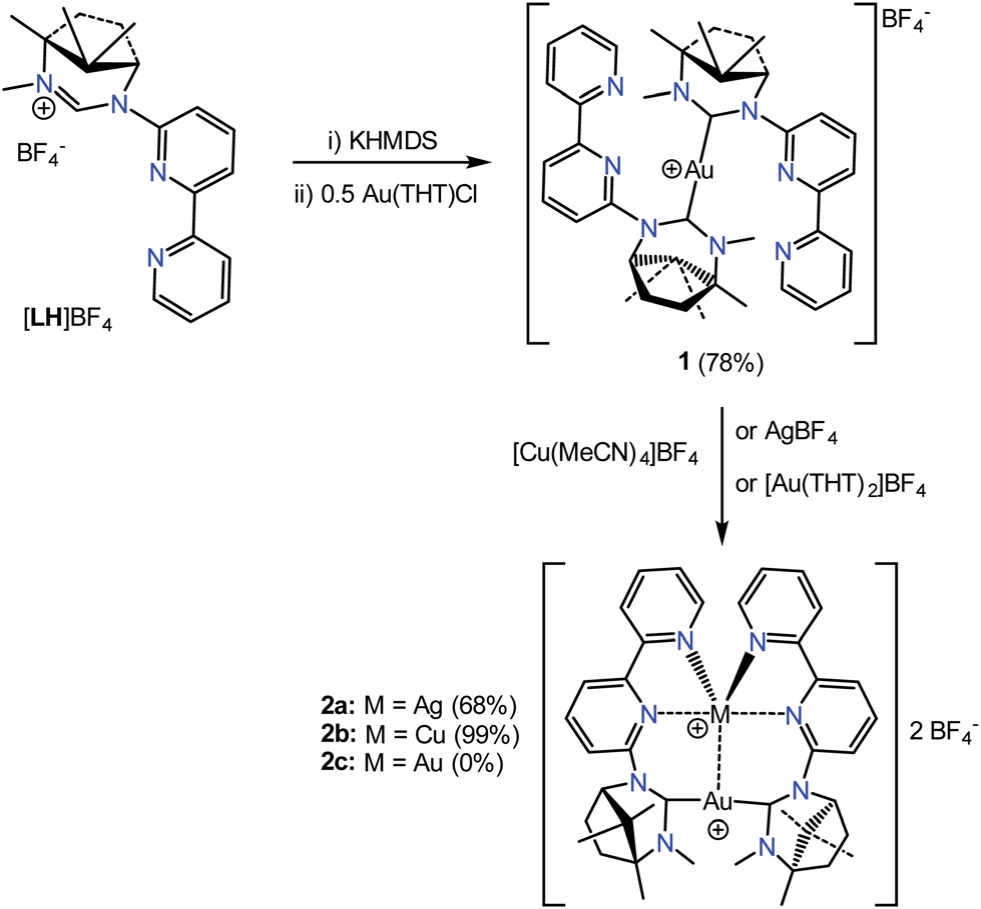
Formation of the mixed metal bimetallic complexes.

The masked ligand synthesis has been described previously.^18^ The ligand has been designed with the latent NHC function at the 6-position to enable controlled formation of discrete bimetallic complexes and encourage, within the context of this article, close M⋯M′ contacts. The starting point of the investigation was the robust [Au(κ^C^-L)_2_]BF_4_ complex 1 which was isolated as a cream solid from the 2 : 1 reaction of the free carbene (generated in solution by treating [LH]BF_4_ with KHMDS in THF at −40 °C) with Au(THT)Cl (Scheme 1). This was based on the rationale that the Au(i) would have little desire to coordinate with the bipy fragment leaving it available for binding the second metal.

The isolated complex 1 proved to be both air- and moisture-stable and showed no sign of decomposition upon exposure to light in the solid state or in solution. Loss of the NCHN proton is evident upon inspection of the ^1^H NMR spectrum where an upfield shift is observed for both the bridgehead hydrogen of the bicyclic ring and the NC*H*_3_ hydrogens compared to their respective positions in the ^1^H NMR spectrum of [LH]BF_4_. These observations are commensurate with the loss of the positive charge on the ligand. The two ligands in [Au(κ^C^-L)_2_]BF_4_ are equivalent as highlighted by the presence of a single set of signals in the ^1^H and ^13^C{^1^H} NMR spectra. This confirms facile rotation about the Au–C bonds in solution on the NMR timeframe. The complex 1 is observed at *m*/*z* = 837.3665 in the mass spectrum (ES+) with a smaller peak at 517.1660 for the mono-ligated [Au(κ^C^-L)]^+^ fragment.

**Fig. 1:**
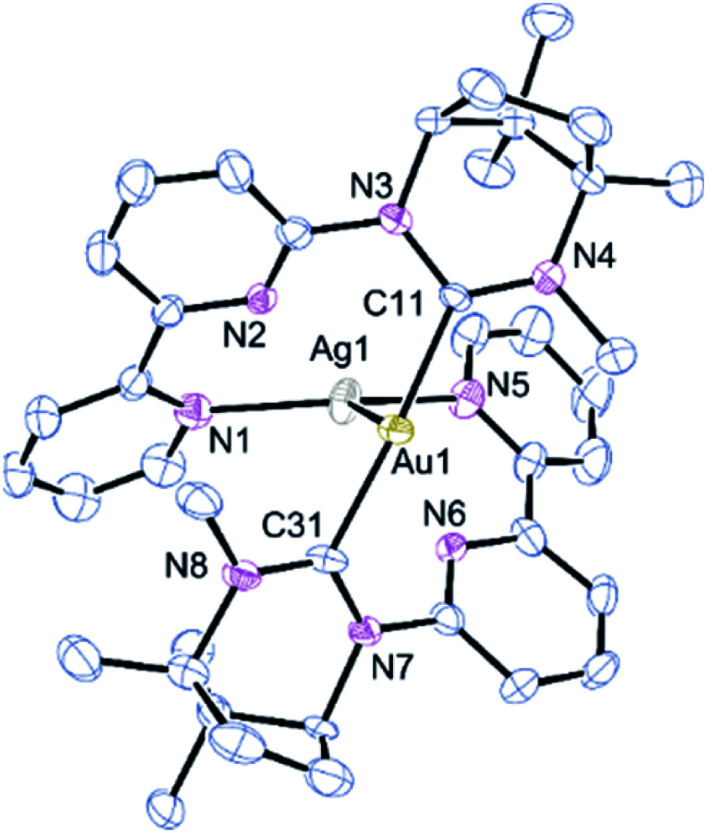
Molecular structure of [AuAg(κ-*C*^Au^,κ^2^-*N*,*N*′^Ag^-1)_2_]^2+^, 2a. All counterions and solvent molecules have been removed for clarity. Selected bond lengths (Å) and angles (°): Au1–Ag1 2.8544(9); Au1–C11 2.031(8); Au1–C31 2.045(9); Ag1–N1 2.262(9); Ag1–N2 2.515(8); Ag1–N5 2.224(8); Ag1–N6 2.674(8); C11–Au1–C31 175.3(4); N1–Ag1–N5 136.5(3); N2–Ag1–N6 129.0(3).

Combining 1 with one equivalent of AgBF_4_ led to the isolation of the bimetallic complex [AuAg(κ-C^Au^,κ^2^-*N*,*N*′^Ag^-1)_2_](BF_4_)_2_, 2a (Scheme 1). The molecular structure of the cation of 2a, as determined by single-crystal X-ray diffraction techniques, is shown in Fig. 1. The Au(i) centre is bound by the two NHC carbon atoms with the Ag(i) ion coordinated to the bipy arms and supported by a metallophilic interaction with the gold. The Ag–Au bond is short at 2.8544(9) Å which compares with values of 2.8359(4), 2.8039(7) and 2.8200(6) Å reported for related pyridine–NHC ligands,^5^ NHC–phosphine hybrid ligands^10^ and unsupported Au–Ag interactions.^1*k*^ The only reported crystal structures of a bipyNHC ligand with silver(i) show only κ-C coordination.^7^ The C–Au–C linkage is close to linear and the coordination geometry about the gold is described as T-shaped when the silver is included as a donor. The Au–C bond lengths (av. 2.039 Å) and N–C–N angles are comparable to those of reported ring-expanded NHC complexes of the same type.^13,19^ The Ag–N bond lengths vary from an average of 2.248(9) Å for the terminal pyridines to 2.538(8) Å for the pyridine rings attached to the NHC units. As these longer Ag–N bonds are shorter than the sum of the van der Waals radii the coordination geometry about the Ag can be described as a heavily distorted trigonal bipyramid with the two more weakly bound nitrogens occupying the apical sites. The Au and remaining two nitrogens define the trigonal plane with an obtuse N–Ag–N angle of 136.5(3)° and two Au–Ag–N angles of 111.8(2) and 110.7(2)° (*Σ* = 359.07°). This necessarily means the arrangement of the NHC ligands about the gold is transoid with respect to the N-substituents.

The integrity of the bimetallic complex is retained in the gas phase as evidenced by HRMS where the parent dication is observed at *m*/*z* = 472.1362. The peak seen at *m*/*z* = 517.1674 corresponds to [Au(κ^C^-L)]^+^ and that at 321.2075 to [LH]^+^. Some broadening is noted for certain resonances in the ^1^H and ^13^C{^1^H} NMR spectra of 2a which suggests a degree of fluxionality in solution and the NC*H*_3_ signal is shifted ∼1 ppm upfield compared to its position in 1, presumably as a consequence of the methyl residing over one or more of the bipy aromatic rings.

Further evidence for the significance of the Au–Ag contact is demonstrated by DFT calculations. The cationic core of 2a is retained after geometry optimisation with only minor changes in Au–Ag and metal–ligand lengths (ESI† for details). Atoms-in-molecules analysis locates a bond critical point in Au–Ag bond (ρc = 0.023 au) with associated bond path. Moreover, a relaxed potential energy scan of Au–Ag distance shows rapid energy loss upon increasing the distance between the Au and Ag atoms to the extent that, the complex containing both two coordinate gold and two coordinate silver and no metallophilic interaction is around 136 kJ mol^−1^ less stable.

The [AuCu(κ-*C*^Au^,κ^2^-*N*,*N*′^Cu^-1)_2_](BF_4_)_2_, 2b was prepared in a similar manner to that of 2a by the 1 : 1 reaction of 1 with [Cu(MeCN)_4_]BF_4_ in (CD_3_)CO. Addition of 1 to the copper precursor produced an immediate colour change from colourless to deep orange-red. Removal of the solvent gave a red solid which, upon examination by ^1^H NMR spectroscopy showed some significant differences to that of 1 highlighting coordination of the Cu(i) centre by the bipy ligands. The main changes in the ^1^H NMR spectrum are seen in the aromatic region where every signal is shifted downfield upon coordination to Cu(i). The doublet for the bridgehead hydrogen has also shifted downfield but the N–CH_3_ methyl has shifted significantly upfield (3.29 ppm in 1, 2.75 ppm in 2b) as has one of the other methyl resonances albeit to a lesser extent. The ^13^C NMR spectrum of 2b also differs significantly from that for 1 with all peaks being shifted downfield relative to their position in the ^13^C{^1^H} NMR spectrum of 1. The HRMS shows the parent dication at *m*/*z* = 450.1482. Despite numerous attempts to produce single crystals of the complex, we were unable to obtain crystals of suitable quality to enable determination of the solid-state molecular structure. Although we do not have definitive empirical data for the structure of the bimetallic Au/Cu complex, theoretical calculations (see below and ESI†) strongly support a similar structure to that established for 2a.

**Fig. 2:**
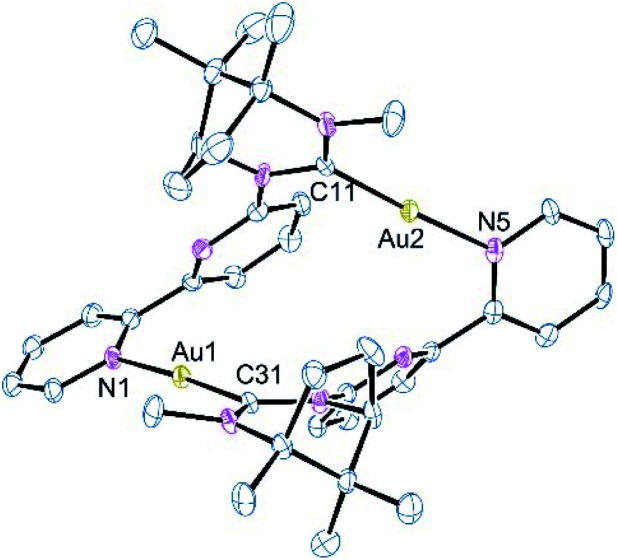
Molecular structure of [AuAu′(κ-C^Au/Au′^,κ^1^-N^Au/Au′^-1)_2_]^2+^, 3. All counterions and solvent molecules have been removed for clarity. Selected bond lengths (Å) and angles (°): Au1–C31 1.989(8); Au1–N1 2.065(6); Au2–C11 1.996(8); Au2–N5 2.060(7); C31–Au1–N1 176.4(3); C11–Au2–N5 176.3(3).

The reaction of 1 with Au(THT)Cl in the presence of one equivalent of AgBF_4_ or with *in situ* generated [Au(THT)_2_]BF_4_ gave, from HRMS analysis, a species containing two ligands and two gold atoms. However, examination of the ^1^H NMR spectrum of the isolated solid showed a mixture of at least two species which proved difficult to separate by crystallisation. Examination of a single blocky crystal by single-crystal X-ray techniques revealed the complex shown in Fig. 2. The structure consists of two gold centres each coordinated by the NHC carbon of one ligand and the terminal pyridine of the bipy of a second ligand; there is no obvious Au⋯Au contact as the distance of 5.498 Å is too great for any metallophilic interaction. The structure has the two pyridyl rings in each ligand skewed at an angle of 45° and fixed in the *R*_a_ configuration about this chiral axis. There appears to be a π-stacking interaction between the two non-coordinated pyridine rings which lie almost exactly over one another even though the centroid–centroid distance is long at 4.00 Å. The Au–C and Au–N bond lengths are typical^20^ and the N–Au–C bonds are close to linear.

While the formulation accords with that expected on the basis of the HRMS the structure is not analogous to 2a and 2b. This unexpected result would suggest that the integrity of 1 has been compromised under the reaction conditions with one NHC donor being transferred to a second Au(i) ion. It is possible that complex 3 has arisen from traces of [Au(κ^C^-L)Cl] in the starting material. However, this was never detected in the starting complex 1 and we were not able to acquire this species from the 1 : 1 reaction of L with [Au(THT)Cl]. In all cases where a 1 : 1 ratio of ligand to [Au(THT)Cl] was adopted, only [Au(κ^C^-L)_2_]BF_4_ was isolated. The fact that NMR analysis shows a mixture of products but the HRMS data shows only a peak corresponding to the formulation [Au_2_(L)_2_]^2+^ does suggest that the mixture observed in solution is a combination of [AuAu′(κ-C^Au^,κ-N,^Au′^-1)_2_](BF_4_)_2_, 2c and [AuAu′(κ-C^Au/Au′^,κ^1^-N^Au/Au′^-1)_2_](BF_4_)_2_, 3. The only other complex that could be isolated on occasion from the attempted preparation of the digold complex through initial halide abstraction from [Au(THT)Cl] with AgBF_4_ was [AuAg(κ-C^Au^,κ^2^-*N*,*N*′^Ag^-1)_2_](BF_4_)_2_, 2a. Hence Ag(i) seems to compete with Au(i) for the nitrogen donors reflecting a preference for binding of Ag(i) over Au(i) in the bimetallics as confirmed by theoretical calculations (see below).

The apparent instability of 2c compared to the analogous Au/Ag and Au/Cu complexes is a consequence of the rigid nature of the ligand which is unable to flex sufficiently to allow for the accommodation of a Au⋯Au fragment. This is compounded by the strong desire for Au(i) to be linear; this would not be possible in a structure akin to that observed for the Au/Ag complex. The instability of 2c is confirmed from calculations which show the most stable form of the digold complex to be one in which the gold atoms are well separated with close to linear coordination geometries. These calculations also show a strong energetic preference for the formation of the mixed metal species with the Au/Ag complex being 63 kJ mol^−1^ more stable than the Au/Au and Ag/Ag combination.

Although calculations show the homo- and hetero-species containing Ag and Cu to be potentially stable, attempts to prepare the necessary [Ag(κ^C^-L)_2_]BF_4_ and [Cu(κ^C^-L)_2_]BF_4_ starting complexes were unsuccessful. All efforts to acquire the discrete mononuclear, bis(NHC) complexes were thwarted by the presence of several different products from which nothing proved tractable. This is likely a result of both these metal ions being more promiscuous in their coordination geometries/numbers. We are currently investigating the extension of the gold chemistry for the inclusion of metals other than Ag and Cu with emphasis on d^6^ transition metals such as Re(i) and Ir(iii) and will report the results of these studies in due course.

## Conclusions

An asymmetric, bipy–NHC hybrid ligand has been prepared and used for the construction of mixed group 11 bimetallic complexes of the type [AuM(κ-*C*^Au^,κ^2^-*N*,*N*′^M^-L)_2_](BF_4_)_2_. Prior formation of the [Au(κ-C-1)_2_](BF_4_) complex is critical to the successful employment of these ligands as coordination flexibility precludes isolation of NHC-bonded Cu or Ag analogues. DFT data confirm the stability of the heterometallic core and the importance of specific Au–Ag bonding within this.

## Author contributions

PDN: conceptualisation, methodology, investigation, synthesis, data acquisition, writing. BMK: X-ray crystallography. JAP: calculations.

## Conflicts of interest

There are no conflicts to declare.

## Supplementary Material

RA-011-D1RA06581E-s001

RA-011-D1RA06581E-s002

## References

[cit1] Campos J. (2020). Nat. Rev. Chem..

[cit2] Pezük L. G., Şen B., Hahn F. E., Türkmen H. (2019). Organometallics.

[cit3] Chen K., Nenzel M. M., Brown T. M., Catalano V. J. (2015). Inorg. Chem..

[cit4] Munro L. B., Catalano V. J. (2014). Eur. J. Inorg. Chem..

[cit5] Catalano V. J., Malwitz M. A., Etogo A. O. (2004). Inorg. Chem..

[cit6] Kaub C., Lebedkin S., Bestgen S., Köppe R., Kappes M. M., Roesky P. W. (2017). Chem. Commun..

[cit7] Deiβler C., Rominger F., Kunz D. (2009). Dalton Trans..

[cit8] Liu B., Pan S., Liu B., Chen W. (2014). Inorg. Chem..

[cit9] Gourlaouen C., Danopoulos A. A., Braunstein P., Ai P. (2016). Inorg. Chem..

[cit10] Ai P., Mauro M., Cola L. D., Danopoulos A. A., Braunstein P. (2016). Angew. Chem., Int. Ed..

[cit11] Mora M., Gimeno M. C., Visbal R. (2019). Chem. Soc. Rev..

[cit12] Huang B., Hu M., Toste F. D. (2020). Trends Chem..

[cit13] Sampford K. R., Carden J. L., Kidner E. B., Berry A., Cavell K. J., Murphy D. M., Kariuki B. M., Newman P. D. (2019). Dalton Trans..

[cit14] Gauthier E. S., Cordier M., Dorcet V., Vanthuyne N., Favereau L., Williams J. A. G., Crassous J. (2021). Eur. J. Org. Chem..

[cit15] Schmidbaur H., Schier A. (2012). Chem. Soc. Rev..

[cit16] Bouché M., Mordan M., Kariuki B. M., Coles S. J., Christensen J., Newman P. D. (2016). Dalton Trans..

[cit17] Rais E., Florke U., Wilhelm R. (2017). Synthesis.

[cit18] Jerwood K., Lowy P., Deeming L., Kariuki B. M., Newman P. D. (2021). Dalton Trans..

[cit19] Phillips N., Trifoin R., Aldridge S. (2014). Chem.–Eur. J..

[cit20] Zhang X., Gu S., Xia Q., Chen W. (2009). J. Organomet. Chem..

