# Supplementary material for: A hybrid bipy–NHC ligand for the construction of group 11 mixed-metal bimetallic complexes
Source: RSC Adv. 2021 Oct 21;11(54):34170–3. doi: 10.1039/d1ra06581e (PMC9042343; doi:10.1039/d1ra06581e)
Supplement: RA-011-D1RA06581E-s001 [file RA-011-D1RA06581E-s001.pdf]

Electronic Supplementary Material (ESI) for RSC Advances.
This journal is © The Royal Society of Chemistry 2021

Supplementary material.

A Hybrid Bipy-NHC Ligand for the Construction of Group 11 Mixed-Metal Bimetallic Complexes

Benson M. Kariuki,[a] James A. Platts[a] and Paul D. Newman[b]*

[†]School of Chemistry, Cardiff University, Cardiff, CF10 3AT, UK.

[‡]Cardiff Catalysis Institute, School of Chemistry, Cardiff University, Cardiff, CF10 3AT, UK.

Contents

NMR and MS data: S2

[Au(κ-*C*-**L**)$_2$]BF$_4$, **1** S2

[AuAg(κ-*C*^{Au},κ^2-*N,N'*^{Ag}-**1**)$_2$](BF$_4$)$_2$, **2a** S5

[AuCu(κ-*C*^{Au},κ^2-*N,N'*^{Cu}-**1**)$_2$](BF$_4$)$_2$, **2b** S7

[AuAu'(κ-*C,N*$^{Au,Au'}$,κ^1-*N*$^{Au,Au'}$-**1**)$_2$](BF$_4$)$_2$, **3** S11

Theoretical calculations of metallophilic interactions S12

Crystallographic data S15

References………………………………………………………………………………………………………S17

[Au(κ-*C*-**L**)$_2$]BF$_4$, **1**.

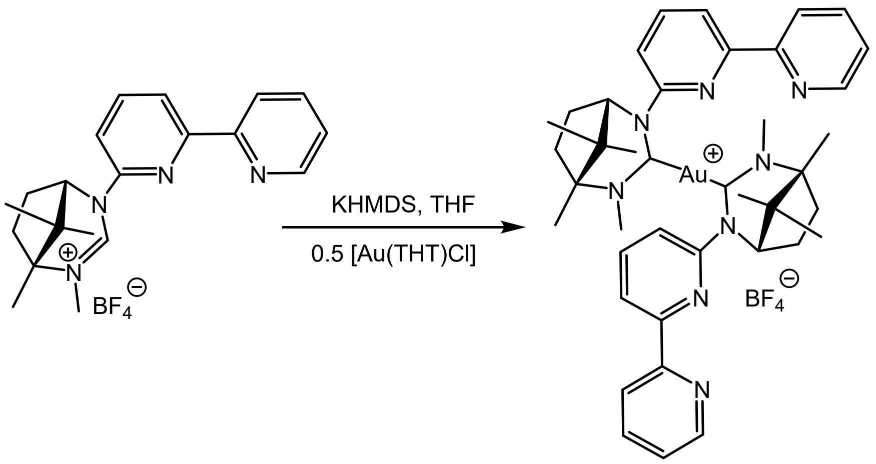

To a stirred suspension of [LH]BF$_4$ (100 mg, 2.45 x 10^{-4} mol) in THF (10 ml) at -40 °C was added, as a solid, KHMDS (59 mg, 2.94 x 10^{-4} mol). The addition of the base caused the salt to dissolve to give an orange solution. After one hour at -40 °C, solid [Au(THT)Cl] (39 mg, 1.22 x 10^{-4} mol) was added and the stirred solution allowed to warm slowly to RT in the absence of light. The volatiles were removed in vacuo and the resultant solid dissolved in CH_2Cl_2 (5 ml) and filtered through a fibreglass filter. After removal of volatiles the residue was crystallised from acetone by vapour-diffusion of pentane to give colourless crystals. Yield = 88 mg (78%).

^{1}H (d$_6$-acetone, 400 MHz): 8.65 (ddd, 4.7, 1.7, 0.9 Hz, 1H), 8.40 (dt, 8.0, 0.9 Hz, 1H), 8.19 (dd, 7.8, 0.8 Hz, 1H), 7.94 (td, 7.7, 1.8 Hz 1H), 7.80 (t, 7.8 Hz, 1H), 7.42 (ddd, 7.6, 6.0, 1.2 Hz, 1H), 7.24 (dd, 7.9, 0.8 Hz, 1H), 3.89 (d, 5.1 Hz, 1H), 3.29 (s, 3H), 2.35-1.80 (m, 4H), 1.26 (s, 3H), 1.05 (s, 3H), 0.94 (s, 3H) ppm. ^{13}C{^{1}H} ($CDCl_3$, 125 MHz): 196.2 (N*C*N), 154.2 (C), 152.5 (C), 152.4 (C), 146.8 (CH), 136.7 (CH), 134.6 (CH), 121.8 (CH), 118.6 (CH), 117.3 (CH), 116.9 (CH), 68.6 (C), 66.8 (CH), 39.0 (C), 38.2 (CH_2), 35.9 (CH_3), 28.7 (CH_2), 19.6 (CH_3), 15.3 (CH_3), 13.3 (CH_3) ppm. HRMS (ES): m/z 837.3665 (calc. 837.3667) [M]$^+$, 100%.

^{1}H NMR spectrum of [Au(κ-*C*-**L**)$_2$]BF$_4$ recorded at 400 MHz in d$_6$-acetone.

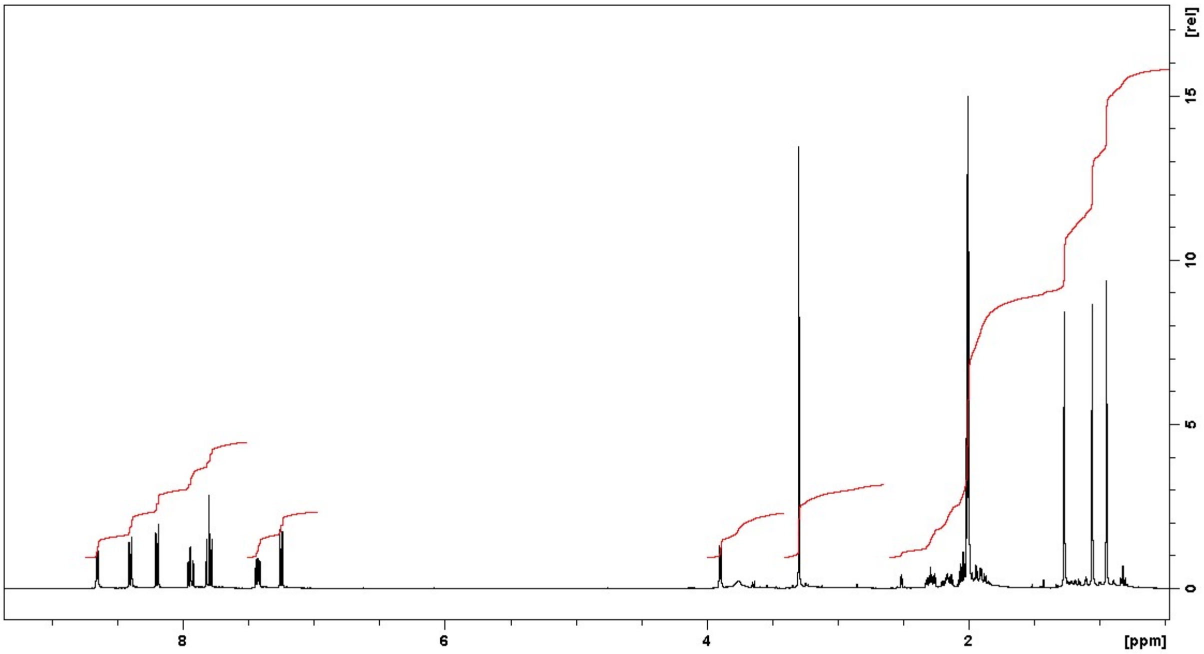

^{13}C NMR spectrum of [Au(κ-*C*-**L**)$_2$]BF_4 recorded at 125 MHz in $CDCl_3$.

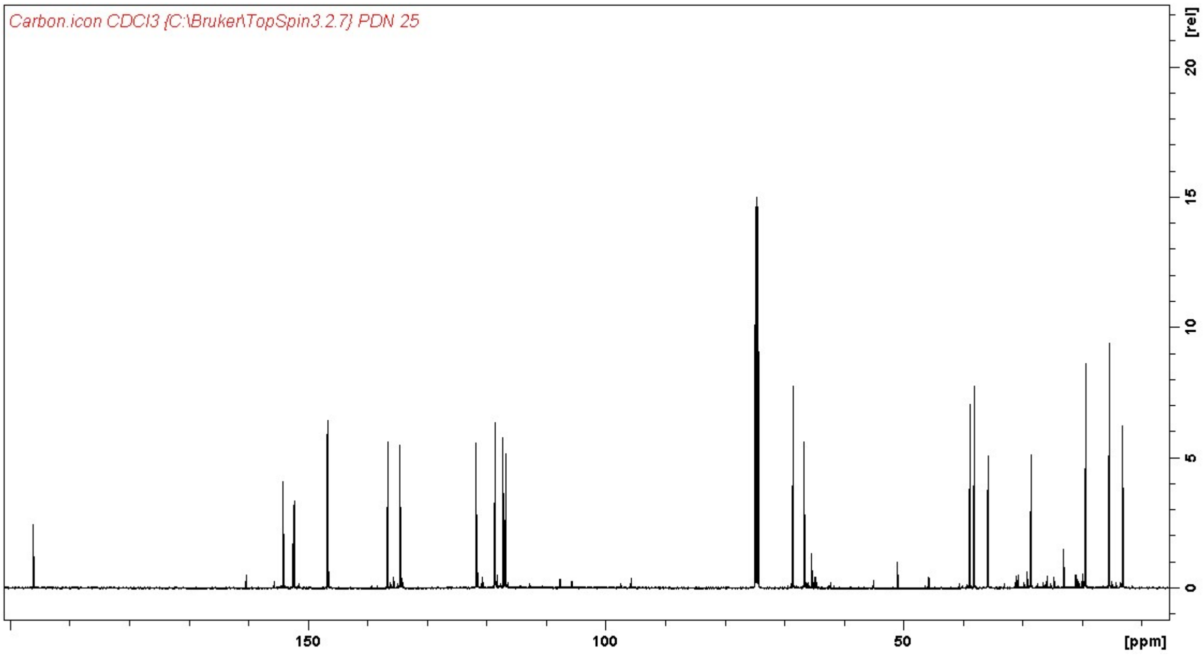

HRMS (ES$^+$) spectrum of [Au(κ-*C*-**L**)$_2$]BF_4.

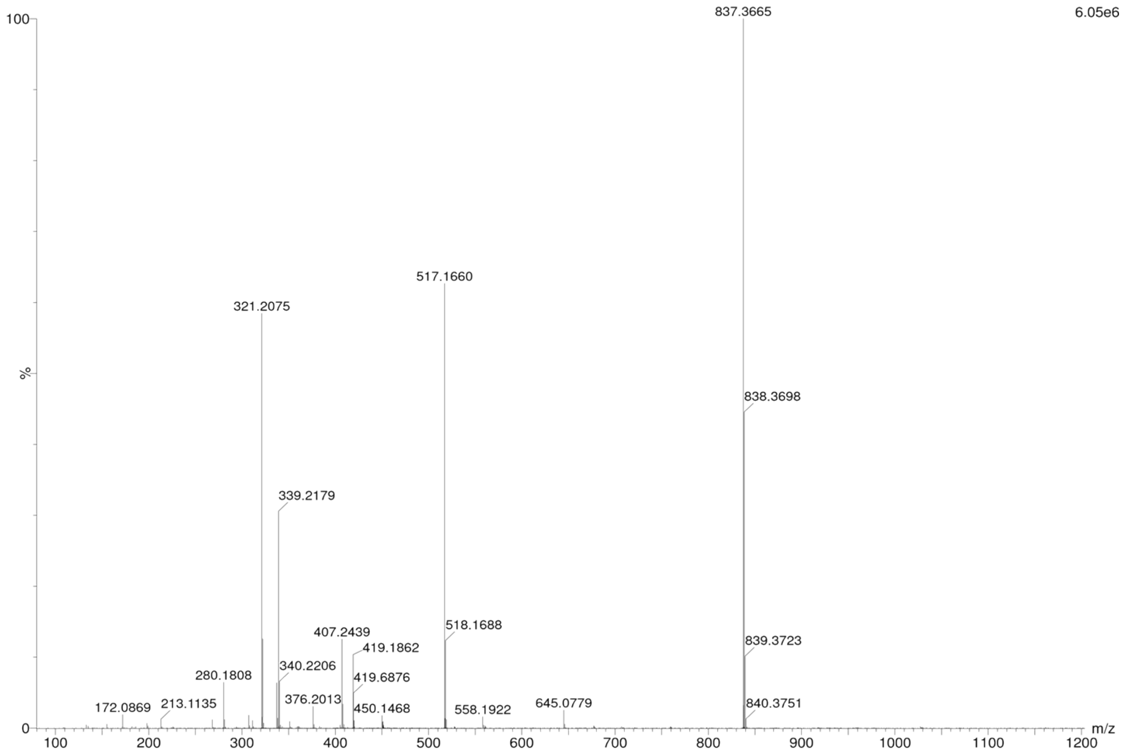

Single Mass Analysis

Tolerance = 5.0 PPM / DBE: min = -1.5, max = 200.0
Element prediction: Off
Number of isotope peaks used for i-FIT = 3

Monoisotopic Mass, Odd and Even Electron Ions
15 formula(e) evaluated with 1 results within limits (up to 50 closest results for each mass)
Elements Used:
C: 0-40 H: 0-48 N: 0-8 197Au: 0-1

Minimum:				-1.5				
Maximum:		5.0	5.0	200.0				
Mass	Calc. Mass	mDa	PPM	DBE	i-FIT	Norm	Conf(%)	Formula
837.3665	837.3667	-0.2	-0.2	21.5	346.4	n/a	n/a	C40 H48 N8 197Au

[AuAg(κ-C^{Au},κ^2-N,N'^{Ag}-**1**)$_2$](BF_4)$_2$, **2a**.

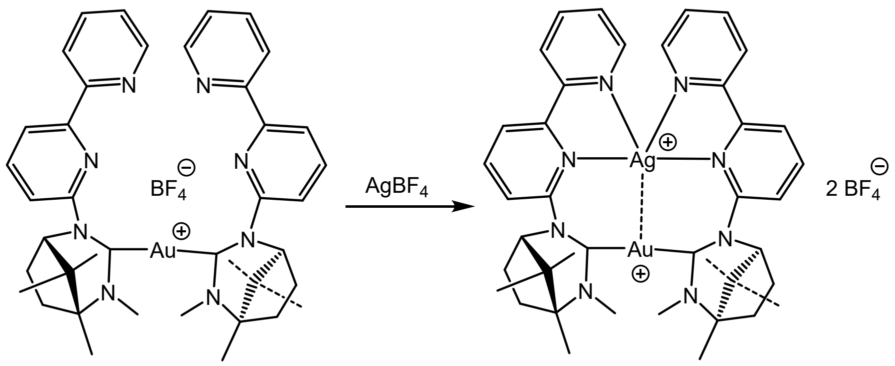

A mixture of [Au(κ-C-**1**)$_2$]BF_4 (20.0 mg, 2.26 x 10^{-5} mol) and $AgBF_4$ (4.4 mg, 2.26 x 10^{-5} mol) were added to d_6-acetone (1.0 ml) and sonicated for ten seconds. The pale yellow solution was isolated from the grey solid and taken to dryness to yield a cream solid. Yield = 68%. Crystals suitable for analysis by single-crystal X-ray crystallography were obtained by vapour diffusion of Et_2O into a solution of the complex in acetone. ^{1}H (CD_3CN, 400 MHz): 8.80 (br, 2H), 8.51 (d, 8.1 Hz, 2H), 8.35 (d, 7.9 Hz, 2H), 8.23 (m, 4H), 7.69 (dd, 6.9, 5.6 Hz, 2H), 7.53 (d, 7.9 Hz, 2H), 3.86 (br, 2H), 2.68 (s br, 6H), 2.20-1.60 (m br, 8H), 1.10 (s br, 9H), 1.04 (s br, 9H) ppm. ^{13}C{^{1}H} (CD_2CN, 150 MHz): 198.0 (N*C*N), 157.4 (C), 151.9 (CH), 151.7 (C), 151.1 (CH), 142.0 (CH), 140.0 (CH), 126.2 (CH), 124.2 (CH), 122.2 (CH), 71.2 (C), 71.2 (CH), 40.8 (C), 40.2 (CH_2), 38.2 (CH_3), 31.4 (CH_2), 20.7 (CH_3), 17.4 (CH_3), 14.6 (CH_3) ppm. HRMS (ES): m/z 473.1365 [M]$^{2+}$, 100%.

^{1}H NMR spectrum of [AuAg(κ-C^{Au},κ^2-N,N'^{Ag}-**1**)$_2$](BF_4)$_2$ recorded at 400 MHz in CD_3CN.

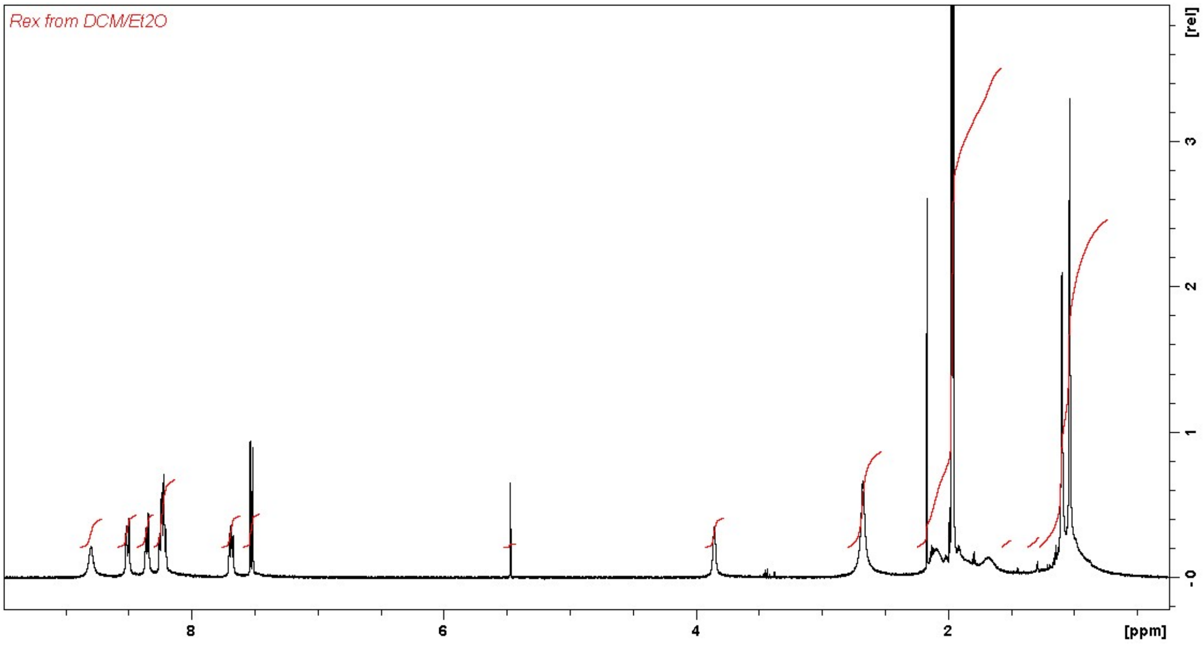

$^{13}C\{^{1}H\}$ NMR spectrum of [AuAg(κ-C^{Au},κ^2-N,N'^{Ag}-**1**)$_2$](BF_4)$_2$ recorded at 150 MHz in CD_3CN.

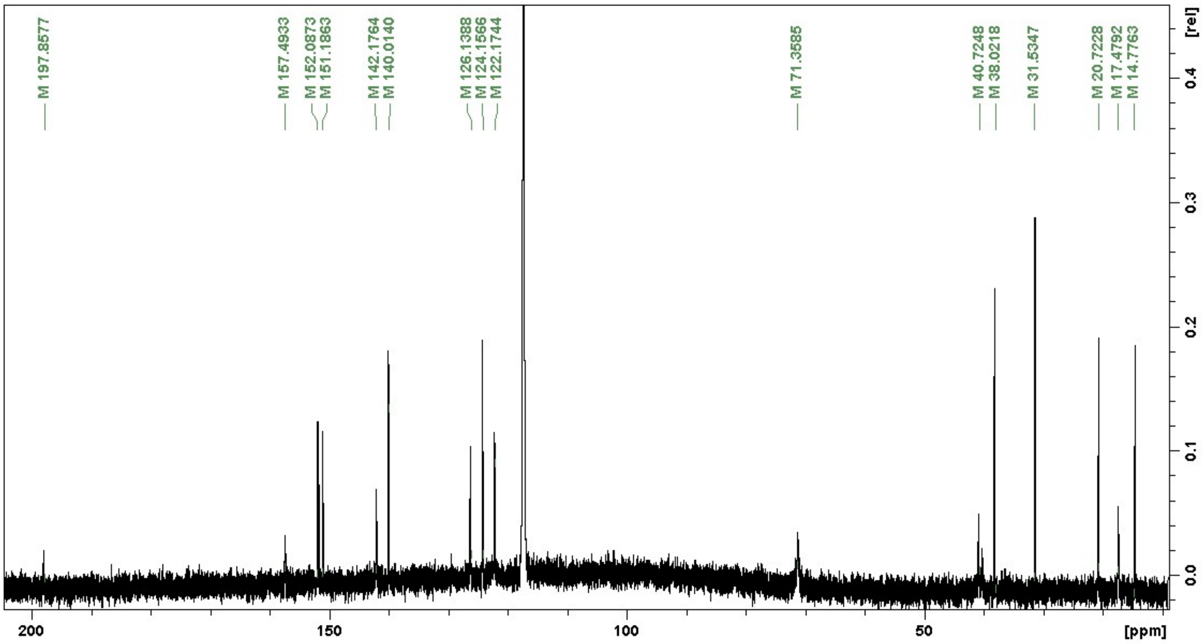

HRMS (ES^+) spectrum of [AuAg(κ-C^{Au},κ^2-N,N'^{Ag}-**1**)$_2$](BF_4)$_2$

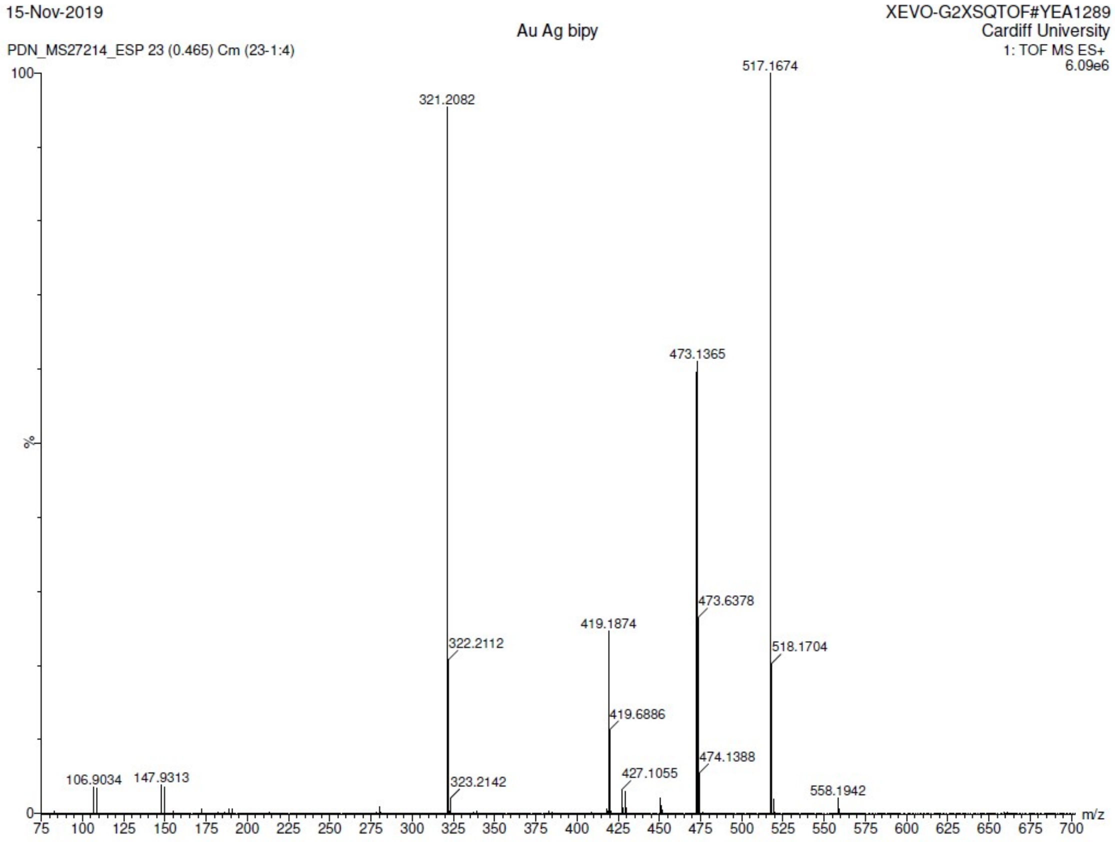

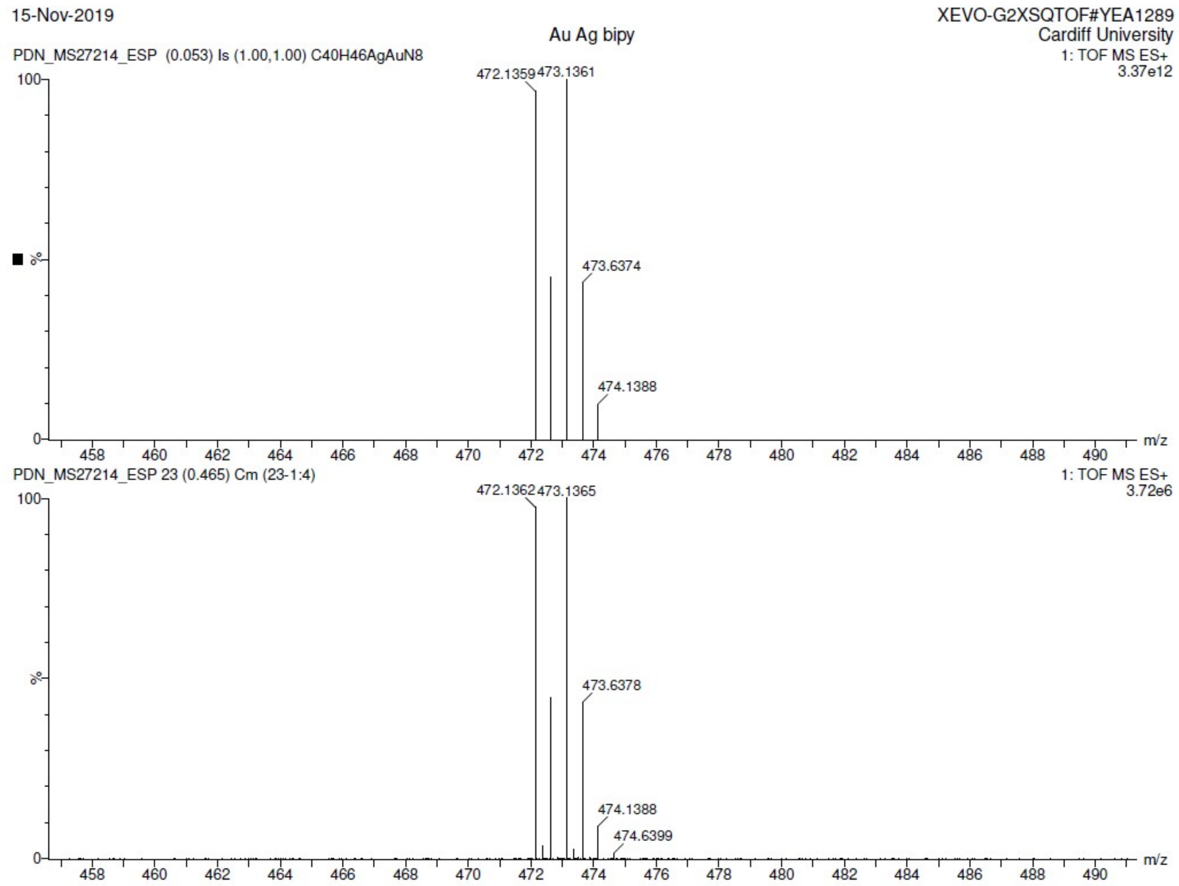

[AuCu(κ-C^{Au},κ^2-*N,N'*^{Cu}-**1**)$_2$](BF$_4$)$_2$, **2b**.

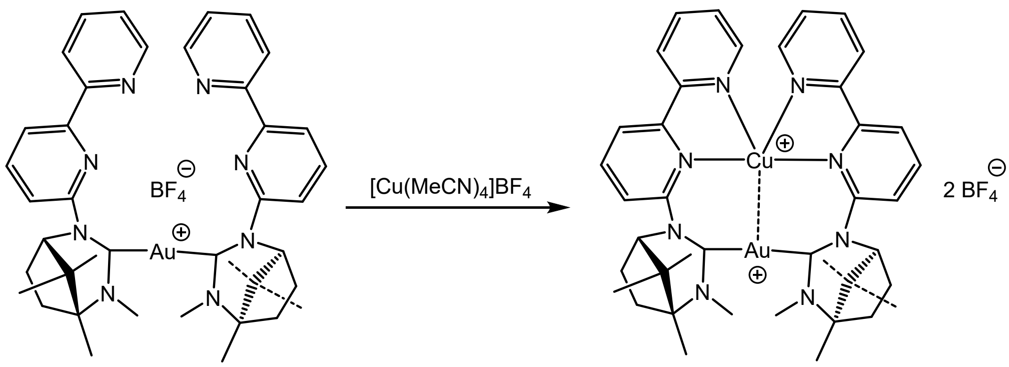

A mixture of [Au(κ-C-**1**)$_2$]BF$_4$ (20.0 mg, 2.26 x 10^{-5} mol) and [Cu(MeCN)$_4$]BF$_4$ (6.8 mg, 2.26 x 10^{-5} mol) were dissolved in acetone (1.0 ml) to give an orange-red solution which was taken to dryness to give an orange-red solid. Yield = quantitative. ^{1}H (d$_6$-acetone, 400 MHz): 8.84 (dd, 5.0, 0.7 Hz, 2H), 8.69 (d, 8.2 Hz, 2H), 8.59 (d, 8.0 Hz, 2H), 8.29 (t, 8.0 Hz, 2H), 8.23 (dt, 8.0, 1.7 Hz, 2H), 7.75 (d, 8.1 Hz, 2H), 7.66 (ddd, 7.4, 5.1, 0.8 Hz, 2H), 4.14 (d, 4.6 Hz, 2H), 2.74 (s, 6H), 2.00-1.50 (m, 8H), 1.09 (s, 6H), 1.05 (s, 6H), 0.99 (s, 6H) ppm. ^{13}C{^{1}H} (d$_6$-acetone, 100 MHz): 199.3 (NCN), 157.8 (C), 152.2 (C), 151.7 (C), 150.4 (CH), 142.8 (CH), 140.2 (CH), 127.8 (CH), 124.2 (CH), 123.7 (CH), 122.0 (CH), 71.7 (C), 71.6 (CH), 41.6 (C), 41.0 (CH$_2$), 39.0 (CH$_3$), 31.8 (CH$_2$), 21.3 (CH$_3$), 17.7 (CH$_3$), 15.4 (CH$_3$) ppm. HRMS (ES): m/z 450.1485 [M]$^{2+}$, 100%.

^{1}H NMR spectrum of [AuCu(κ-*C*^{Au},κ^{2}-*N,N'*^{Cu}-**1**)$_2$](BF$_4$)$_2$ recorded at 400 MHz in d$_6$-acetone.

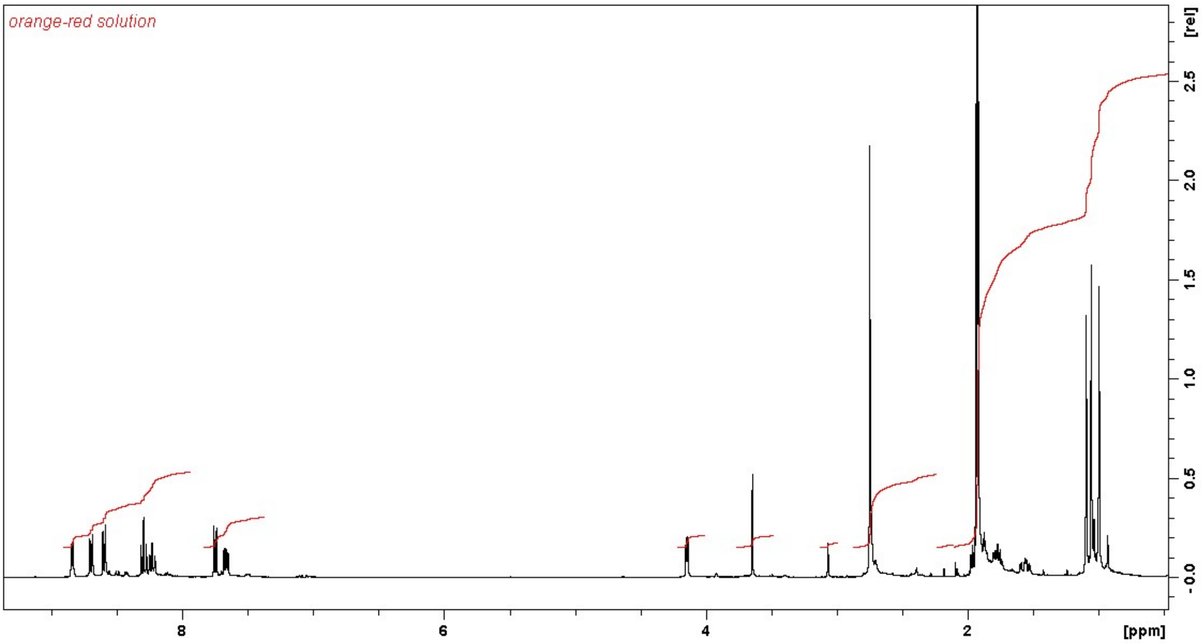

^{13}C NMR spectrum of [AuCu(κ-*C*^{Au},κ^{2}-*N,N'*^{Cu}-**1**)$_2$](BF$_4$)$_2$ recorded at 100 MHz in d$_6$-acetone.

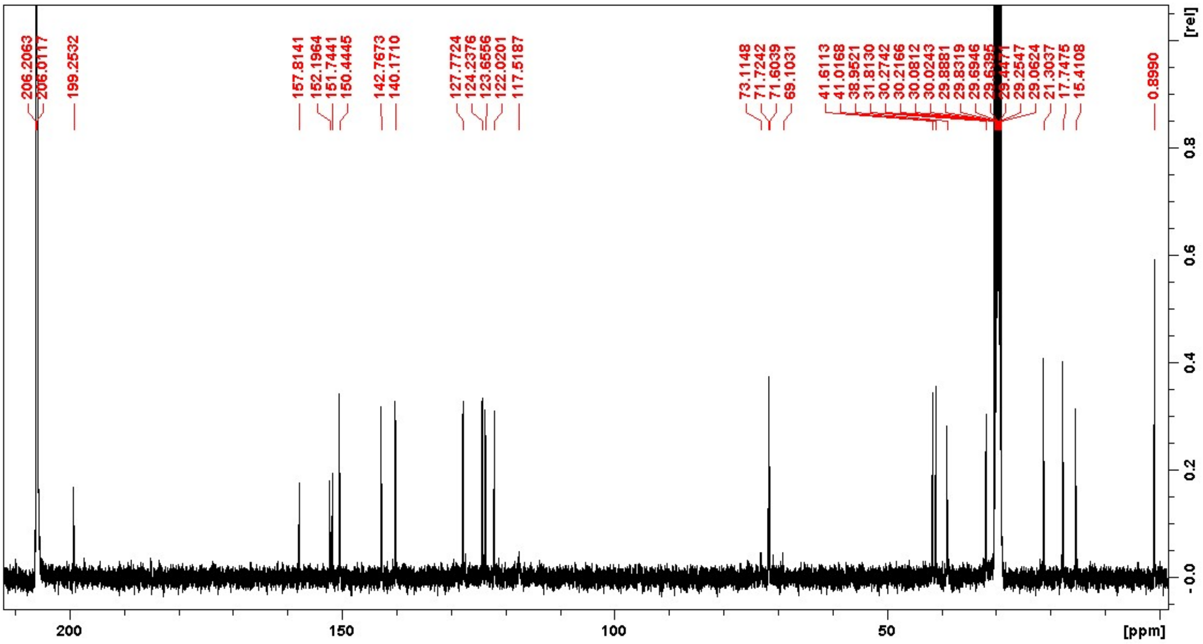

HRMS (ES^{+}) spectrum of [AuCu(κ-*C*^{Au},κ^{2}-*N,N'*^{Cu}-**1**)$_2$](BF$_4$)$_2$

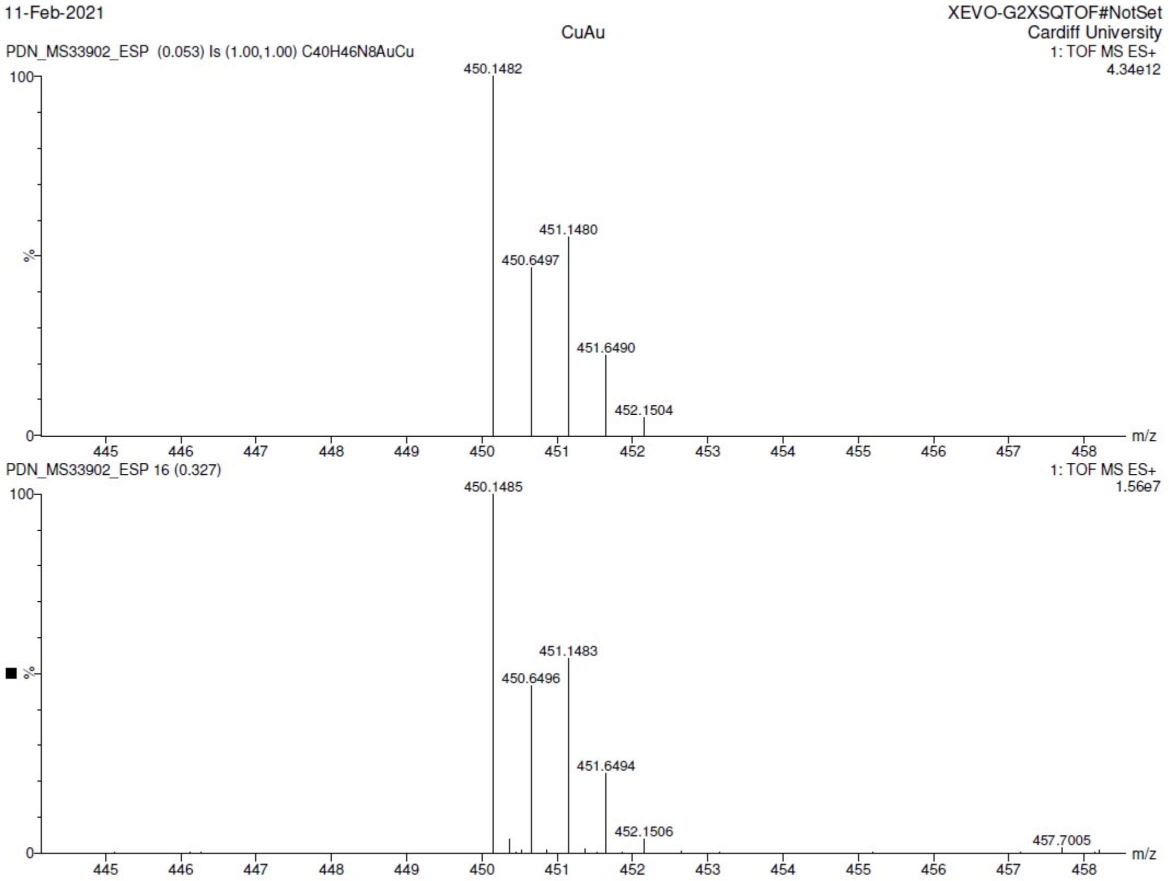

11-Feb-2021
CuAu
XEVO-G2XSQTOF#NotSet
Cardiff University
PDN_MS33902_ESP (0.053) Is (1.00,1.00) C40H46N8AuCu
1: TOF MS ES+
4.34e12
450.1482
451.1480
450.6497
451.6490
452.1504
m/z
PDN_MS33902_ESP 16 (0.327)
1: TOF MS ES+
1.56e7
450.1485
451.1483
450.6496
451.6494
452.1506
457.7005
m/z

Attempted preparation of [AuAu(κ-C^{Au},κ^2-N,N'^{Au}-**1**)$_2$](BF_4)$_2$, **2c**.

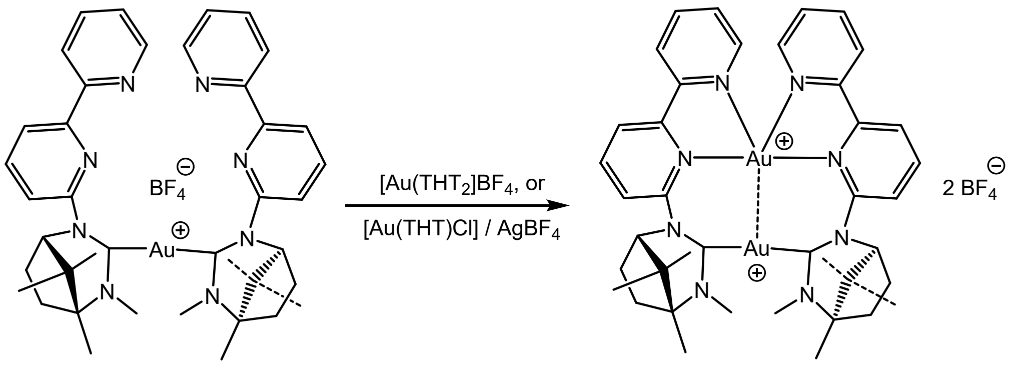

In a typical procedure, a solution of $AgBF_4$ (12 mg, 6.10 x 10^{-5} mol) and THT (18 mg, 1.93 x 10^{-4} mol) in CH_2Cl_2 (5 ml) was stirred for 2 hours in the absence of light whereupon [Au(THT)Cl] (18 mg, 5.65 x 10^{-5} mol) was added as a solid. After stirring for a further 1 hour in the dark, the mixture was filtered into a stirred solution of [Au(κ-C-**1**)$_2$]BF_4 (50.0 mg, 5.65 x 10^{-5} mol) in CH_2Cl_2. After stirring for a further 1 hour in the dark, the solution was filtered and all volatiles removed in *vacuo*. The resultant tan solid was triturated with diethyl ether, filtered and dried in *vacuo*. All attempts to obtain a pure compound by recrystallisation from various solvents were unsuccessful. Only on a single occasion were a small number of crystals obtained by vapour diffusion of pentane into an acetone solution of the mixture. These proved to be [AuAu'(κ-$C^{Au/Au'}$,κ^1-$N^{Au/Au'}$-**1**)$_2$](BF_4)$_2$, **3**. Decomposition was often observed as evidenced by the deposition of dark solids. Changes to the procedure described above included using AgOTf or $AgPF_6$, isolating the [Au(THT)$_2$]BF_4 before the second step (this is not recommended as this material is quite sensitive) and reversing the addition of the final step but none of these changes proved successful.

^{1}H NMR spectrum of the solid resulting from the attempted synthesis of **2c** recorded at 400 MHz in d_6-acetone. This is the best quality sample that could be obtained.

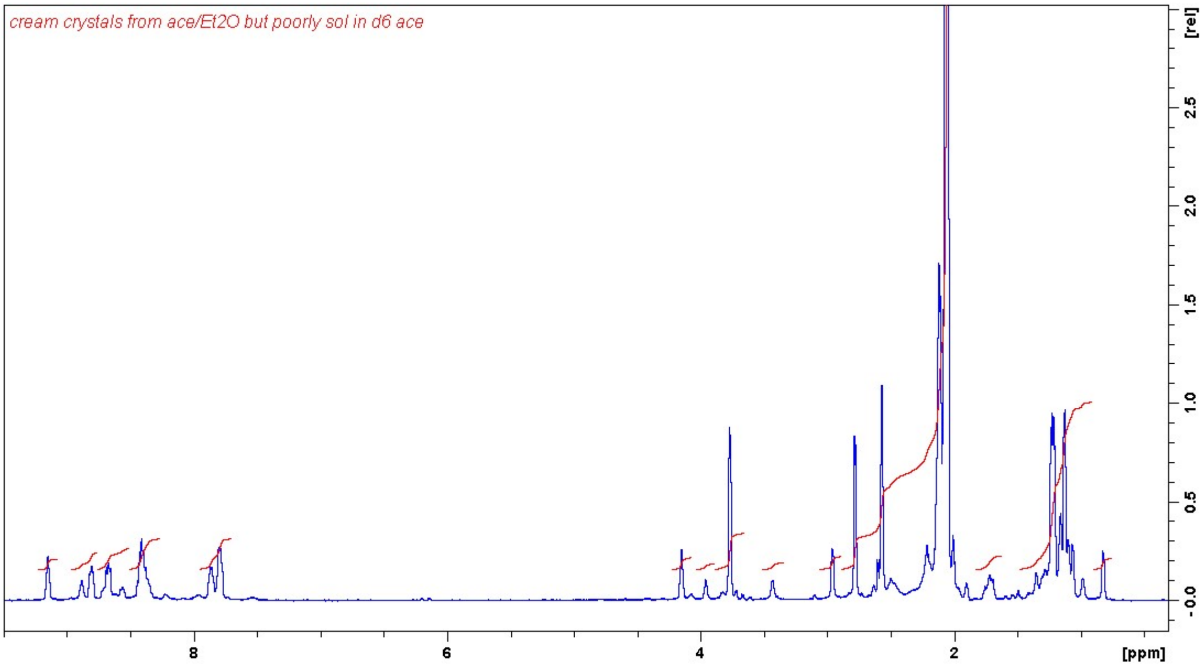

HRMS (ES+) spectrum showing the dication [AuAu'(κ-$C^{Au,Au'}$,κ^1-$N^{Au,Au'}$-**1**)$_2$]$^{2+}$

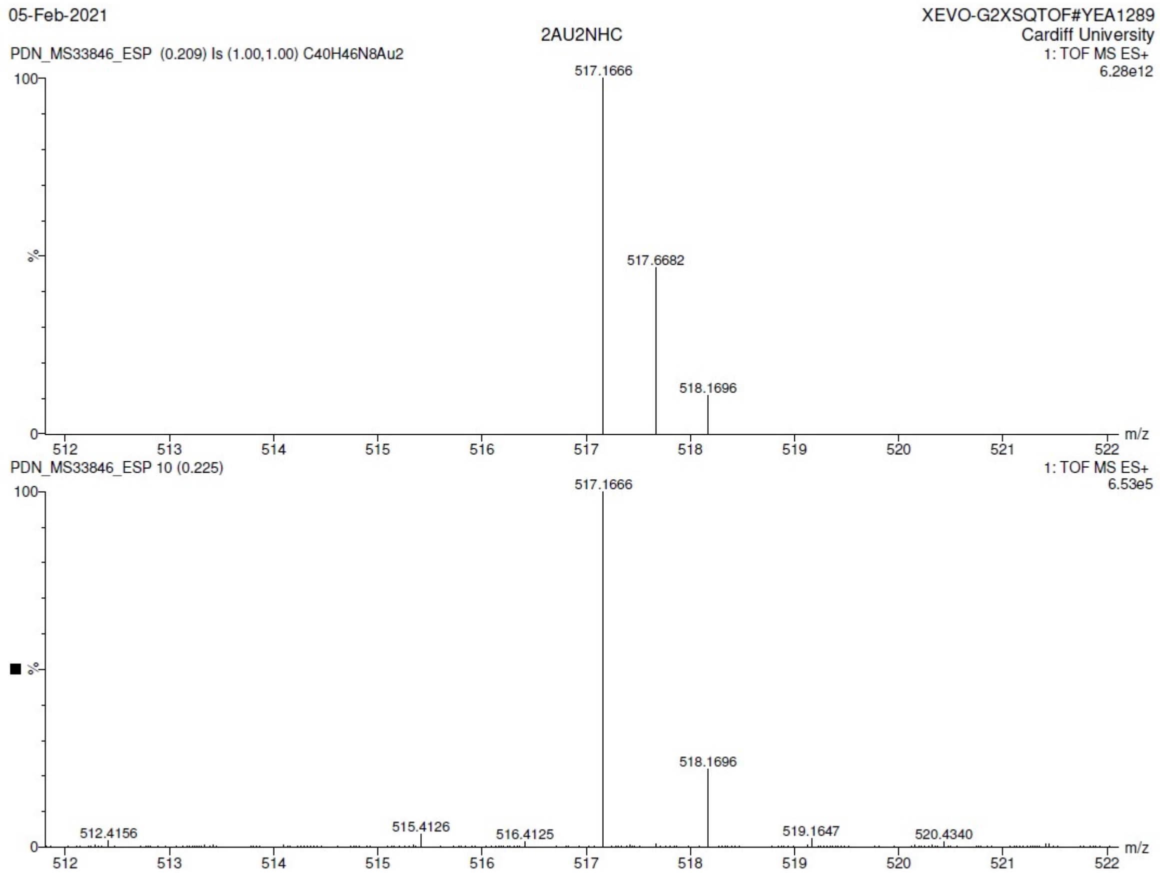

Theoretical details

All DFT calculations were performed in the Gaussian09 suite (rev D.01). PBE0/SDD-6-31G(d) was selected as the optimum method for further study based on comparison with the X-ray structure of **2a** (see below). All compounds were fully optimized without symmetry constraints, and confirmed as minima by harmonic frequency calculation. Relaxed potential energy scan of **2a** along Ag-Au used 50 steps of 0.1 Å starting from the optimized geometry. Hypothetical homo-metallic dimers were constructed from the optimized geometry of **2a** and fully optimized at the same level. Atoms in Molecules analysis used the AIMAll package.

Comparison of selected geometrical details, all with SDD basis/ECP on Ag and Au, 6-31G(d) on all remaining atoms

	Ag-Au	Ag-C	C-Ag-C	Ag-N
M06-2X	3.015	2.088	166.9	2.351, 2.557
PBE0	2.896	2.068	175.5	2.299, 2.501
PBE0-D	2.970	2.065	164.3	2.266, 2.476

Total energies of hetero- and homo-metallic dimers (au)

Au-Ag	-2270.710317
Au-Cu	-2321.041941
Au-Au	-2259.472652
Ag-Ag	-2281.899856
Cu-Cu	-2382.570095
Ag-Cu	-2332.234772

Relative energies of hetero- and homo-metallic dimers (kJ mol^{-1})

2*Au-Ag	Au-Au + Ag-Ag	-63.18
2*Au-Cu	Au-Au + Cu-Cu	-54.00
2*Ag-Cu	Ag-Ag + Cu-Cu	+0.53

Electron density at selected bond critical points in **2a** (au)

Ag-Au	0.0228
Ag-C	0.1229
Ag-N	0.0514, 0.0335
Au…N	0.0142

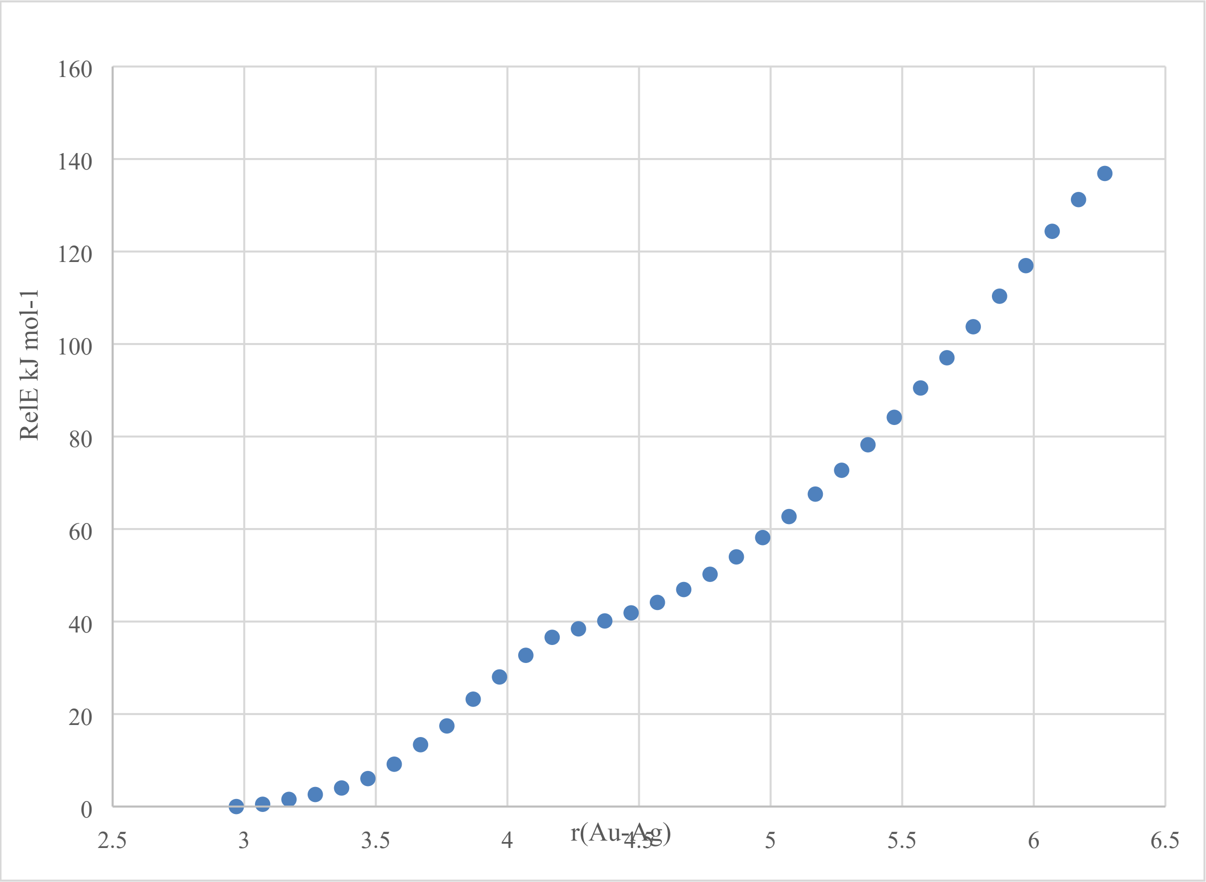

Molecular graph of **2a**

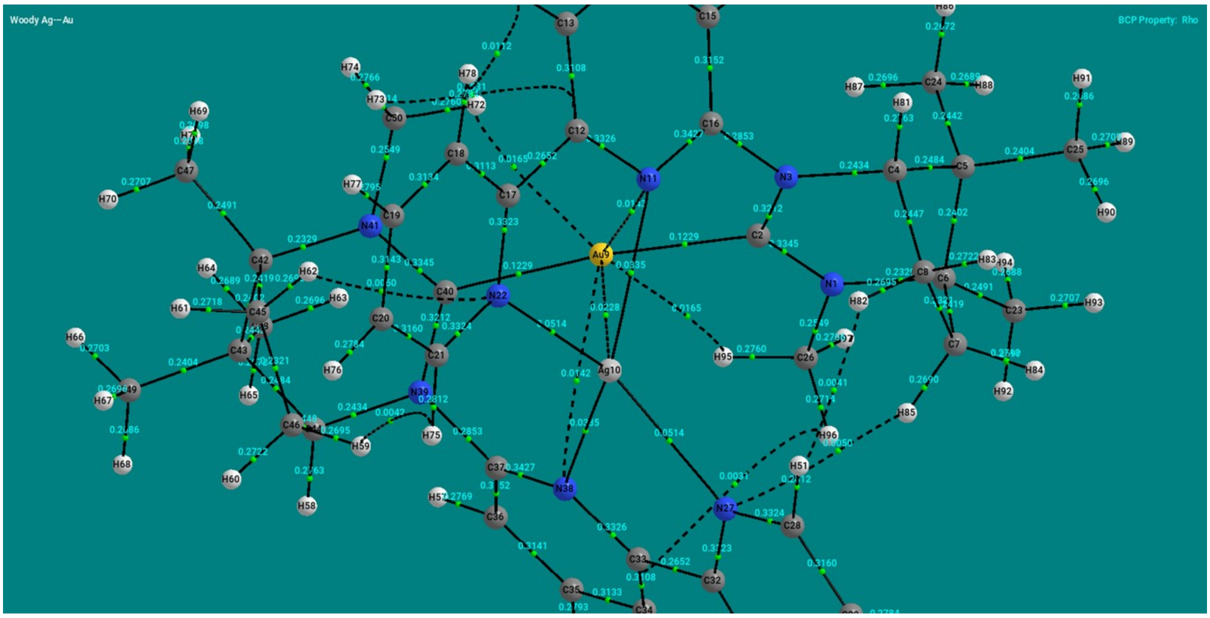

Crystallographic data.
Crystal structure determination

Single-crystal XRD data were collected on an Agilent SupaNova Dual Atlas diffractometer with a mirror monochromator using either Cu (λ = 1.5418 Å) or Mo (λ = 0.7107 Å) radiation. Sample temperature was controlled using an Oxford Cryosystems cooling apparatus. Crystal structures were solved and refined using SHELXS and refined using SHELXL.[1] Non-hydrogen atoms were refined with anisotropic displacement parameters. Hydrogen atoms were inserted in idealized positions, and a riding model was used with Uiso set at 1.2 or 1.5 times the value of Ueq for the atom to which they are bonded.

Table S1. Crystal data and structure refinement for **2** (pdn1903b), and **4** (pdn2002)

Compound (Identification code)	**2a** (pdn1903b)	**3** (pdn2002)
CCDC reference	2106666	2106665
Empirical formula	$C_{40}H_{47.5}B_2F_8N_8AgAu$	$C_{47}H_{60}Au_2BF_7N_8O_5S$
Formula weight	1118.81	1386.83
Temperature /K	200(2)	293(2)
Wavelength /Å	0.71073	0.71073
Crystal system	Monoclinic	Triclinic
Space group	$P2_1$	P_1
a/Å	12.5502(6)	9.2214(3)
b/Å	26.5936(6)	11.7385(4)
c/Å	13.7021(5)	13.0330(4)
α/°	90	72.325(3)
β/°	114.813(5)	75.768(3)
γ/°	90	86.988(3)
Volume/Å^3	4151.0(3)	1302.54(8)
Z	4	1
Density (calculated)/ Mgm^{-3}	1.790	1.768
Absorption coefficient/ mm^{-1}	4.079	5.742
S1Crystal size/ mm^3	0.520 x 0.257 x 0.053	0.229 x 0.173 x 0.154
Reflections collected	19872	12209
Independent reflections	17003	10912
R(int)	0.0392	0.0349
Data / restraints / parameters	19872 / 235 / 1143	12209 / 153 / 691
Goodness-of-fit on F^2	1.040	1.023
R1, wR2 [I>2σ(I)]	0.0397, 0.0833	0.0330, 0.0623
R1, wR2 (all data)	0.0519, 0.0821	0.0407, 0.0663
Absolute structure parameter	-0.005(4)	-0.012(5)
Largest diff. peak and hole e.Å^{-3}	1.468 and -1.781	0.937 and -0.859

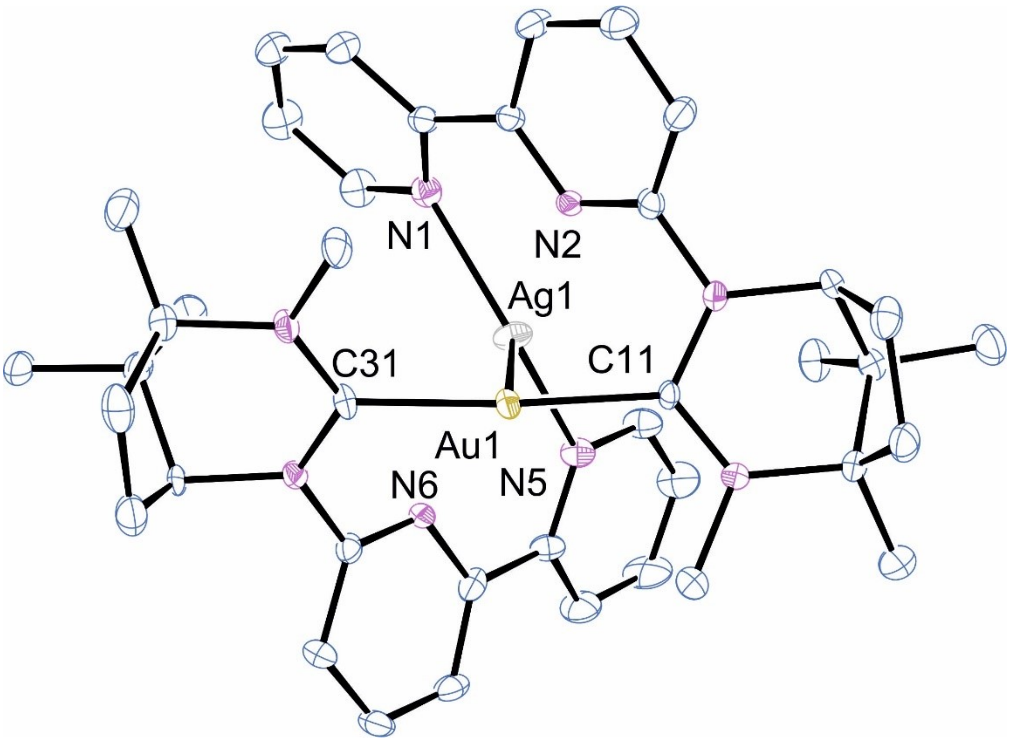

Fig 1: A 30% probability Ortep[3] represenation of complex **2** with hydrogen atoms, solvent molecules and counterion omitted.

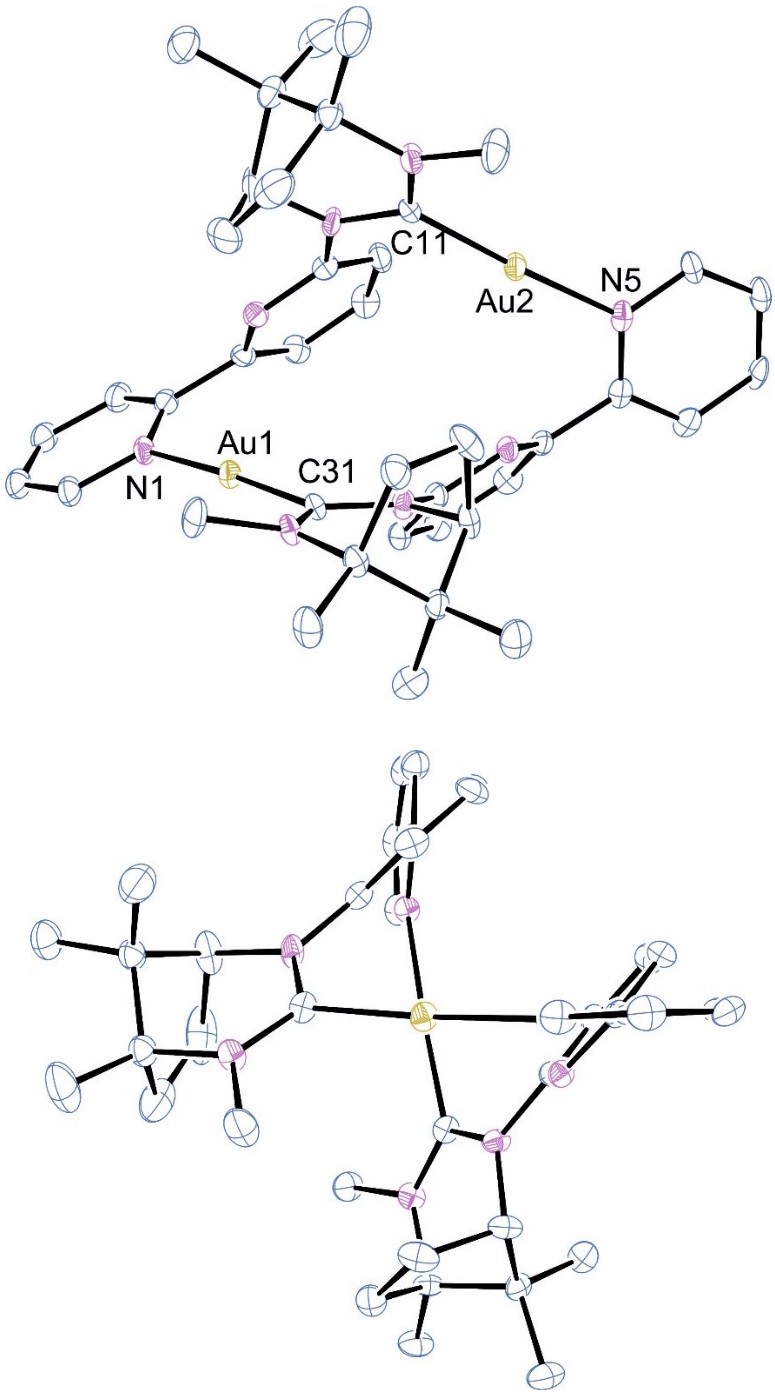

Figure 2: Two Ortep[3] views of the complex cation **4** (the lower one viewed down the Au-Au axis) at 30% probability with hydrogen atoms, solvent molecules and counterion omitted for clarity.

References

1) Sheldrick, G. M. A short history of SHELX *Acta Crystallogr. A* 2008, **64**, 112-122.

2) M. Uzarewicz-Baig, M. Koppenwallner, S. Tabassum and R. Wilhelm, *Appl. Organometal. Chem.*, 2014, **28**, 552-558.

3) L. J. Farrugia, *J. Appl. Cryst.* 2012, **45**, 849-854.
